# Cost-effectiveness analysis of capecitabine maintenance therapy plus best supportive care vs. best supportive care alone as first-line treatment of newly diagnosed metastatic nasopharyngeal carcinoma

**DOI:** 10.3389/fpubh.2022.1086393

**Published:** 2023-01-26

**Authors:** Jiaqi Han, Xiaomeng Lan, Kun Tian, Xi Shen, Jinlan He, Nianyong Chen

**Affiliations:** ^1^Department of Head and Neck Oncology and Department of Radiation Oncology, Cancer Center and Laboratory of Single Cell Research and Liquid Biopsy, West China Hospital, Sichuan University, Chengdu, China; ^2^West China School of Public Health and West China Fourth Hospital, Sichuan University, Chengdu, China; ^3^Department of Urology, Institute of Urology, West China Hospital, Sichuan University, Chengdu, China

**Keywords:** nasopharyngeal carcinoma, cost-effectiveness, capecitabine, maintenance therapy, real-world data

## Abstract

**Objectives:**

Maintenance therapy with capecitabine after induction chemotherapy for patients with newly diagnosed metastatic nasopharyngeal carcinoma (mNPC) has been confirmed to be effective. This study aimed to evaluate the cost-effectiveness of capecitabine as maintenance therapy for patients with mNPC from the Chinese payers' perspective.

**Methods:**

Markov model was conducted to simulate the disease progress and evaluated the economic and health outcomes of capecitabine maintenance plus best-supported care (CBSC) or best-supported care (BSC) alone for patients with mNPC. Survival data were derived from the NCT02460419 clinical trial. Costs and utilities were obtained from the standard fee database and published literature. Measured outcomes were total costs, quality-adjusted life-years (QALYs), life-years (LYs), incremental cost-utility ratios (ICURs), incremental cost-effectiveness ratios (ICERs), incremental net monetary benefit (INMB), and incremental net-health benefit (INHB). Sensitivity analyses were performed to assess model robustness. Additional subgroup cost-effectiveness analyses were accomplished.

**Results:**

Throughout the course of the disease, the CBSC group provide an incremental cost of $9 734 and additional 1.16 QALYs (1.56 LYs) compared with the BSC group, resulting in an ICUR of $8 391/QALY and ICER of $6 240/LY. Moreover, the INHB was 0.89 QALYs, and the INMB was $32 034 at the willingness-to-pay threshold of $36 007/QALY. Subgroup analyses revealed that CBSC presented a positive trend of gaining an INHB in all subgroups compared with the BSC group. The results of sensitivity analyses supported the robustness of our model.

**Conclusion:**

Compared with BSC, after induction chemotherapy, CBSC as a first-line treatment was cost-effective for newly diagnosed mNPC. These results suggest capecitabine maintenance therapy after induction chemotherapy as a new option for patients with newly diagnosed mNPC.

## 1. Introduction

Nasopharyngeal carcinoma (NPC) is an epithelial carcinoma with distinct geographical distribution and is characterized by distinct geographical distribution. According to the Global cancer statistics in 2020, almost 80,000 deaths due to NPC are reported annually most frequently in southern China, Southeastern Asia, and North Africa (1, 2). NPC is an asymptomatic, intrinsically invasive disease, which results in 60–70% of patients being diagnosed with advanced stages, and approximately 10% of patients present with metastases (3, 4). Moreover, there is still a significant percentage of patients who develop distant metastases, becoming a leading cause of treatment failure and a major health concern (5, 6). Thus, developing new treatment options for cancer metastasis is urgently necessary.

Usually, as recommended by the guidelines, platinum-based chemotherapy is the first-line treatment for patients with metastatic nasopharyngeal carcinoma (mNPC) (7, 8). The median progression-free survival (PFS) was 5.0–7.0 months for patients receiving chemotherapy alone (9–11). Recent two clinical trials CAPTAIN-1st and JUPITER-02 have confirmed that combination therapy with chemotherapy and immunotherapy in the first-line treatment for mNPC improves the median PFS to over 10 months (12, 13). However, chemoimmunotherapy improves the therapeutic effect, its high price also represents a substantial financial burden to our society (14, 15). Moreover, high drug prices can lead to reduced adherence in countries where patients have to contribute to treatment costs (16).

Therefore, new therapeutic strategies are needed with stronger efficacy, less expensive, and more readily available. Accumulating evidence suggests that tolerable maintenance of low-dose chemotherapy prolongs the progression-free interval for patients without disease progression after first-line treatment (17–20). Capecitabine, an orally administered fluoropyrimidine used widely as low-dose monotherapy to prevent a recurrence (21, 22). Capecitabine is converted to fluorouracil in tumors without complications related to central venous catheterization, improving compliance and convenience. Results from recent studies have assessed and confirmed the effectiveness of capecitabine as maintenance therapy in breast cancer, metastatic colorectal cancer, as well as resected biliary tract cancer (22–24). Recently, a clinical trial also showed superiority in metronomic capecitabine as adjuvant therapy for patients with locoregionally advanced NPC (25).

In NPC, moreover, a phase 3 randomized clinical trial (NCT02460419) provided evidence supporting the efficacy of capecitabine maintenance therapy as a first-line treatment for mNPC (26). This trial randomized patients with newly diagnosed mNPC who achieved disease control after 4–6 cycles of induction chemotherapy to receive either capecitabine maintenance therapy plus best supported care (CBSC) or best supported care (BSC) alone (26). Patients in the CBSC group had a significantly higher median PFS survival compared to the BSC group (35.9 vs. 8.2 mo, HR 0.44, 95% CI 0.26–0.74, *P* = 0.002) (26). The CBSC group showed higher objective response rates (25 vs. 11.5%) and a longer median duration of response than BSC group (26).

These results suggest capecitabine maintenance therapy after induction chemotherapy as a new option for patients with newly diagnosed mNPC and will be suggested by the 2022 Chinese Society of Clinical Oncology (CSCO) guidelines. Chemoimmunotherapy treatments, which were currently recommended for patients with mNPC by the CSCO clinical guideline, however, its high price often causes a significant economic burden in China. In contrast, capecitabine maintenance therapy has lower drug prices with better patient compliance. Therefore, further detailed cost-effectiveness evaluation on the capecitabine maintenance therapy in mNPC is necessary for policymakers, suppliers, and patients to make a rational decision. This study aimed to compare the cost-effectiveness of CBSC and BSC as first-line treatments after induction chemotherapy for patients with mNPC from the Chinese payers' perspective.

## 2. Materials and methods

### 2.1. Model construction

**Figure 1:**
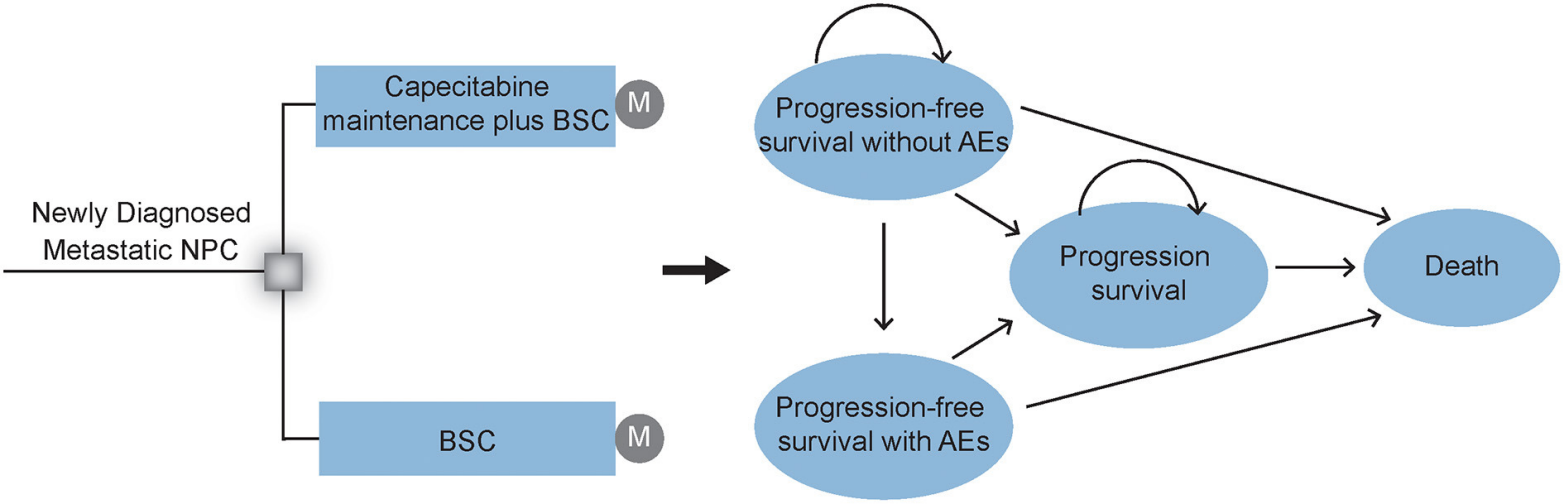
Markov model structure. Markov model structure and Markov states are used to evaluating the economic and health outcomes of CBSC and BSC groups as a first-line treatment after induction chemotherapy for patients with mNPC. After initial treatment, patients could experience a response with or without AEs, or experience the disease progression until death. NPC, nasopharyngeal carcinoma; CBSC, Capecitabine maintenance therapy plus best supported care group; BSC, best supported care; AEs, adverse events.

Comprehensive mathematical Markov model was established to evaluate the economic and health outcomes of adding capecitabine maintenance therapy after induction chemotherapy for patients with newly diagnosed mNPC (Figure 1). We simulated a hypothetical cohort of populations with similar characteristics as those patients enrolled in the NCT02460419 clinical trial (26) (Supplementary Table 1). Eligible patients achieved disease control after 4–6 cycles of paclitaxel (150 mg/m^2^), cisplatin (60 mg/m^2^), and capecitabine (1 000 mg/m^2^ orally twice daily on days 1 to 14) every 21 days. Then they were randomly assigned to two groups in our model according to the first-line treatments: (1) BSC group: provide appropriate palliative care to reduce symptoms and improve quality of life to the greatest extent possible; (2) CBSC group: capecitabine maintenance (1 000 mg/m^2^ orally twice daily on days 1 to 14 of 21-day cycle) for a maximum of 2 years plus BSC treatment until disease progression or intolerable toxicity. Supplementary Table 2 details the drug dosage and schedule.

The Markov model structure used in this analysis was based on previously conducted studies and other economic models. However, the input data and group of the Markov model in this analysis were designed by ourselves. In addition, our Markov model assumed that in each Markov cycle, a patient is always in one exclusive health state. Further, the property of the Markov model was “memoryless” in a mathematical sense. In other words, transitions between the different states depend only on the current state rather than the previous state, is stochastic game.

The Markov model was conducted by using the TreeAge, version 2019 (TreeAge Software, Inc.). As shown in Figure 1, this Markov survival model was composed of 4 exclusive health states to model the disease progress: progression-free survival (PFS) without adverse events (AEs), PFS with AEs, progression disease (PD), and death. During the PFS health state, patients would achieve a response and continue to recieve different first-line therapies, either with or without adverse events (AEs) until progression or unacceptable AEs. All groups received second-line subsequent treatment after progression. The model terminates when all patients die of the disease. Consistent with the treatment cycle, each model cycle represents 3 weeks with a lifetime horizon. Moreover, 3% annual discount rate and half-cycle correlation were adopted for cost and survival estimates (27).

### 2.2. Model transition and survival estimates

Patients in the model transitioned between health states due to the calculated transition probabilities from PFS and OS Kaplan-Meier curves of the NCT02460419 clinical trial (26). The GetData Graph Digitizer software was used to attain the data points to the date of the last follow-up from the PFS and OS curves using the process designated by Hoyle et al. (28). Next, the data were used to fit flexible parametric survival models, including the Exponential, Weibull, Log-logistic, Lognormal, and Gompertz models using the R software. The Log-logistic models provided a good fit for all curves in the two groups according to the visual fit, clinical rationality, and statistical goodness-of-fit (Akaike's information criterion and Bayesian information criterion) (29). The parameters of the Log-logistic distributions, detailed statistical fitting results, and fitting curves in both groups are shown in Supplementary Table 3 and Supplementary Figure 1. The disease-cause mortality rate of transitioning to death was estimated from the OS curves in the NCT02460419 trial, while the other-cause mortality rate was estimated from the recently published Chinese life table (Supplementary Table 4) (30).

### 2.3. Cost inputs

The direct medical costs considered were drug costs, best supportive care, radiation therapy costs, administration, management of severe AEs, laboratory tests, and imaging. Additionally, disease-caused death costs and other-caused death costs were included. The unit costs, such as the price per drug were available from the real-world cost database of West China Hospital, 2022. The prices were recorded in Chinese yuan and then converted into US dollars at an exchange rate of 2022 (1 US dollar = 6.7467 Chinese yuan, August 1 2022) (31). Drug dosage, administration route, medication schedule, and rates of serious AEs in both groups were consistent with the NCT02460419 clinical trial (26). The median medication costs were calculated using the mean weight of 65.0 kg and body surface area of 1.79 m^2^ (32). Further details of the calculation of specific medication costs are available in Supplementary Table 2.

During the first-line treatment, management costs on 3 or 4 grade drug-related AEs were included by multiplying the cost derived from published literature (32, 33) by incidence rates obtained from the NCT02460419 clinical trial (26). In addition, subsequent therapy strategies and their proportions for disease progression were gained from the NCT02460419 clinical trial (26). Costs used for the model analysis are listed in Table 1.

**Table 1:** Model parameters: Baseline values, ranges, and distributions.

**Variable**	**Baseline value**	**Range**		**References**	**Distribution (parameters)**
		**Minimum**	**Maximum**		
OS survival model with CBSC	λ = 0.006220, γ = 1.070002	–	–	(26)	–
OS survival model with BSC	λ = 0.0051219, γ = 1.3690167	–	–	(26)	–
PFS survival model with CBSC	λ = 0.034595, γ = 0.840056	–	–	(26)	–
PFS survival model with BSC	λ = 0.11712, γ = 0.80840	–	–	(26)	–
**Background mortality rate**	Age-specific			(30)	–
**CBSC Concomitant therapy proportion**					
Locoregional radiotherapy	0.528	0.4224	0.6336	(26)	Beta (0.528,0.472)
Bisphosphonates	0.512	0.4096	0.6144	(26)	Beta (0.512, 0.488)
**BSC Concomitant therapy proportion**					
Locoregional radiotherapy	0.476	0.3808	0.5712	(26)	Beta (0.476, 0.524)
Bisphosphonates	0.488	0.3904	0.5856	(26)	Beta 0.488, 0.512)
**CBSC Subsequent therapy proportion**					
Chemotherapy	0.608	0.4864	0.7296	(26)	Beta (0.608, 0.392)
Radiotherapy	0.087	0.0696	0.1044	(26)	Beta (0.087, 0.913)
Immunotherapy	0.087	0.0696	0.1044	(26)	Beta (0.087, 0.913)
**BSC Subsequent therapy proportion**					
Chemotherapy	0.467	0.3736	0.5604	(26)	Beta (0.467, 0.533)
Radiotherapy	0.054	0.0432	0.0648	(26)	Beta (0.054, 0.946)
Immunotherapy	0.162	0.1296	0.1944	(26)	Beta (0.162, 0.838)
**CBSC AEs incidence (Grade 3 or 4)**					
Anemia	0.120	0.096	0.144	(26)	Beta (6, 44)
Neutropenia	0.020	0.016	0.024	(26)	Beta (1,49)
Thrombocytopenia	0.020	0.016	0.024	(26)	Beta (1, 49)
Hand-foot syndrome	0.100	0.08	0.12	(26)	Beta (5, 45)
Nausea and vomiting	0.060	0.048	0.072	(26)	Beta (3, 47)
Mucositis	0.040	0.032	0.048	(26)	Beta (2, 48)
Fatigue	0.040	0.032	0.048	(26)	Beta (2, 48)
**BSC AEs incidence (Grade 3 or 4)**					
Anemia	0.021	0.0168	0.0252	(26)	Beta (1, 47)
Neutropenia	0.000	0.000	0.000	(26)	—
Thrombocytopenia	0.000	0.000	0.000	(26)	—
Hand-foot syndrome	0.000	0.000	0.000	(26)	—
Nausea and vomiting	0.000	0.000	0.000	(26)	—
Mucositis	0.000	0.000	0.000	(26)	—
Fatigue	0.000	0.000	0.000	(26)	—
**Drug cost per dosage unit, $**					
Capecitabine	1.91	1.528	2.292	Local database	Gamma (96.04, 50.28)
Paclitaxel	27.94	22.352	33.528	Local database	Gamma (96.04, 3.44)
Cisplatin	3.00	2.4	3.6	Local database	Gamma (96.04, 32.01)
Bisphosphonates	250.47	200.376	300.564	Local database	Gamma (96.04, 0.383)
Docetaxel	75.56	60.448	90.672	Local database	Gamma (96.04, 1.27)
Gemcitabine	26.05	20.84	31.26	Local database	Gamma (96.04, 3.69)
Irinotecan	159.20	127.36	191.04	Local database	Gamma (96.04, 0.60)
Camrelizumab	458.93	367.144	550.716	Local database	Gamma (96.04, 0.21)
Toripalimab	142.01	113.608	170.412	Local database	Gamma (96.04, 0.68)
Nivolumab	1451.41	1161.128	1741.692	Local database	Gamma (96.04, 0.07)
Pembrolizumab	2808.46	2246.768	3370.152	Local database	Gamma (96.04, 0.03)
**Radiotherapy-related cost, $**	3690.64	2952.51	4428.77	Local database	Gamma (96.04, 0.03)
**Body weight (kg)**	65	32.5	97.5	(32)	Gamma (15.37, 0.24)
**Body surface area(m** ^**2**^ **)**	1.72	1.376	2.064	(32)	Gamma (96.04, 55.84)
**Best supportive care, $**	274.00	219.20	328.80	(32)	Gamma (96.04, 0.35)
**Imaging/Surveillance, $**	176.49	141.19	211.79	(34)	Gamma (96.03, 0.54)
**Laboratory test, $**	82.59	66.07	99.11	(34)	Gamma (96.02, 1.16)
**Discount rate, %**	3	0	8	(27)	Beta (0.03, 0.97)
**AEs cost, $**					
Anemia	6434.00	5147.2	7720.8	(32)	Gamma (96.04, 0.01)
Neutropenia	466.00	372.8	559.2	(32)	Gamma (96.04, 0.21)
Thrombocytopenia	3551.70	2841.36	4262.04	(32)	Gamma (96.04, 0.03)
Hand-foot syndrome	773.64	618.912	928.368	Local database	Gamma (86.37, 0.12)
Nausea and vomiting	44.30	35.44	53.16	(33)	Gamma (96.04, 2.17)
Mucositis	3719	2975.2	4462.8	(32)	Gamma (96.04, 0.03)
Fatigue	107.01	85.608	128.412	(33)	Gamma (96.04, 0. 98)
**Utility**					
PFS	0.760	0.608	0.912	(34)	Beta (0.76, 0.24)
PD	0.350	0.280	0.420	(34)	Beta (0.35, 0.65)
Death	0	0	0	–	–

OS, overall survival; PFS, progression-free survival; CBSC, capecitabine maintenance therapy plus best supportive care; BSC, capecitabine maintenance therapy plus best supportive care; AEs, adverse events; PD, progression disease.

### 2.4. Utility inputs

Quality of life was modeled using health state utility weights. Each health state was assigned with a health utility preference on a scale of 0 (death) to 1 (perfect health). Due to the lack of mature quality-of-life data in the NCT02460419 trial, estimates for the utilities in PFS and PD states were derived from previous literature (34). In addition, the disutility due to the drug-related AEs was considered in the model (35). Detailed information is mentioned in Table 1.

### 2.5. Base case analysis

The main measured outcomes were total costs, quality-adjusted life-years (QALYs), life-years (LYs), incremental cost-utility ratios (ICURs), and incremental cost-effectiveness ratios (ICERs). These calculations are presented in the following equations:


$$\begin{array}{l} ICUR=\frac{\left(C1-C0\right)}{\left(U1-U0\right)}=\;\frac{C}{U};\\ ICER=\frac{\left(C1-C0\right)}{\left(E1-E0\right)}=\;\frac{C}{E}, \end{array}$$

Where C, U, and E represent the total costs, QALYs, and LYs of CBSC (1) or BSC (0), respectively. Based on the recommendation of the China guidelines for pharmacoeconomic evaluations and the World Health Organization (WHO), we used three times the gross domestic product (GDP) per capita ($36 007, in 2022 US$) (31, 36) in China indicator for willingness-to-pay (WTP) threshold (37, 38). The ICERs—incremental costs divided by incremental QALYs gained—were calculated to be compared with a WTP threshold of $36 007/ QALY in two groups.

Moreover, we also calculated the incremental monetary benefit (INMB) and incremental net-health benefit (INHB) based on the following methods:


$$\begin{array}{l} INMB=\left(U1-U0\right)\times WTP-\left(C1-C0\right)=\Delta U\times WTP-\Delta C;\\ INHB=\left(U1-U0\right)-\frac{\left(C1-C0\right)}{WTP}=\Delta U-\;\frac{\Delta C}{WTP}, \end{array}$$

Where U represent the QALYs, and C represent the total costs of CBSC (1) or BSC (0), respectively.

Additionally, we considered the cost-effectiveness in subgroups using the publishing subgroup analysis data in the NCT02460419 trial (26). Patients were stratified according to age, smoking, disease stage, metastases type, liver metastases, lung metastases, bone metastases, response, and Epstein-Barr virus (EBV) DNA copy numbers. All subgroups were assumed to have the same data apart from the available PFS HRs in this model because of lacking sufficient data.

### 2.6. Sensitivity analysis

Sensitivity analyses were completed to ascertain the robustness of the model and uncertainty of the variables impact on the results. A series of one-way sensitivity analyses were carried out with all parameters varied within reasonable bounds of ±20% from their baseline values, as shown in Table 1 (39). Furthermore, probabilistic sensitivity analyses were conducted to estimate variations in inputs changed simultaneously with a specific pattern of statistical distributions as revealed in Table 1 by conducting 10 000 Monte Carlo estimations (40).

## 3. Results

### 3.1. Baseline results and subgroup results

Markov transition probabilities between each state were calculated based on Log logistic model until death, which is accessible in Supplementary Figure 2. Within a lifetime horizon, the baseline results in each group are summarized in Table 2.

**Table 2:** Baseline results.

**Strategy**	**CBSC**	**BSC**
**Cost, $**		
Progression-free survival	51 721	28 603
Overall	105 676	95 942
**QALYs**		
Progression-free survival	2.58	1.44
Overall	3.37	2.21
LYs	5.64	4.08
ICUR, $/QALY ^a^	8 391	NA
ICER, $/LY ^a^	6 240	NA
INHB, QALY ^a^	0.89	NA
INMB, $ ^a^	32 034	NA

BSC, best supportive care; CBSC, Capecitabine maintenance plus BSC; ICUR, incremental cost-utility ratio; ICER, incremental cost-effectiveness ratio; INHB, incremental net health benefit; INMB, incremental net monetary benefit; NA, not applicable; QALY, quality-adjusted life-years. ^a^Compared with BSC.

In the PFS state, patients in the CBSC group provided an additional 1.14 PFS QALYs with an incremental cost of $23 118 compared with the BSC group. Throughout the course of the disease, patients with newly diagnosed mNPC received CBSC providing an additional cost of $9 734 and incremental 1.16 QALYs (1.56 LYs) in the comparison with the BSC, resulting in an ICUR of $8 391/QALY, which was less than the WTP threshold suggesting that the CBSC was cost-effective from the payer's perspective. Moreover, the INHB was 0.89 QALYs, and the INMB was $32 034 at the WTP threshold of $36 007/QALY in the entire disease course, indicating that the CBSC was cost-effective.

**Figure 2:**
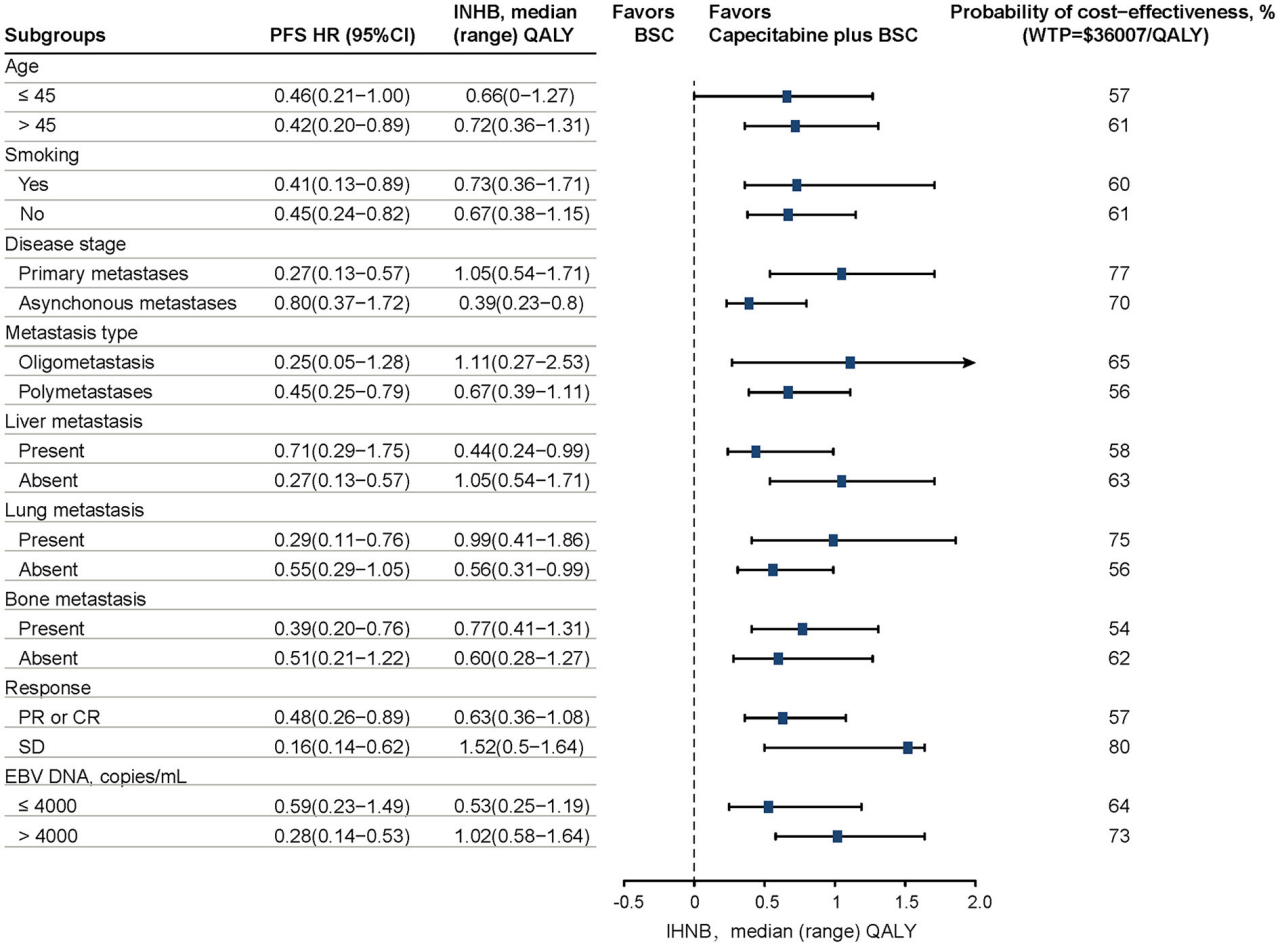
Results of prespecified subgroup analyses in INHBs and probabilities of cost-effectiveness by varying HRs of PFS. The vertical dashed line indicates the point of no effect (INHB = 0), the blue squares indicate the median INHBs, and the black solid bars indicate the ranges of INHB adjusted by HRs. PFS, progression-free survival; HR, hazard ratio; INHB, incremental net health benefit; BSC, best-supported care; WTP, willingness-to-pay; QALY, quality-adjusted life-year.

Prespecified subgroup analyses revealed that compared with the BSC group, CBSC presented a positive trend of gaining an INHB and a high probability of cost-effectiveness at the WTP threshold of $36 007/QALY in all subgroups. For these subgroups, the INHBs concerning the health benefit ranked the subgroup from high to low as stable disease response to first-line chemotherapy [1.52, range (0.5–1.64)], oligometastatic type [1.11, range (0.27–2.53)], primary metastasis stage [1.05, range (0.54–1.71)], and absent of liver metastasis [1.05, range (0.54–1.71)]. Refer to Figure 2 for additional details.

### 3.2. Sensitivity analysis results

**Figure 3:**
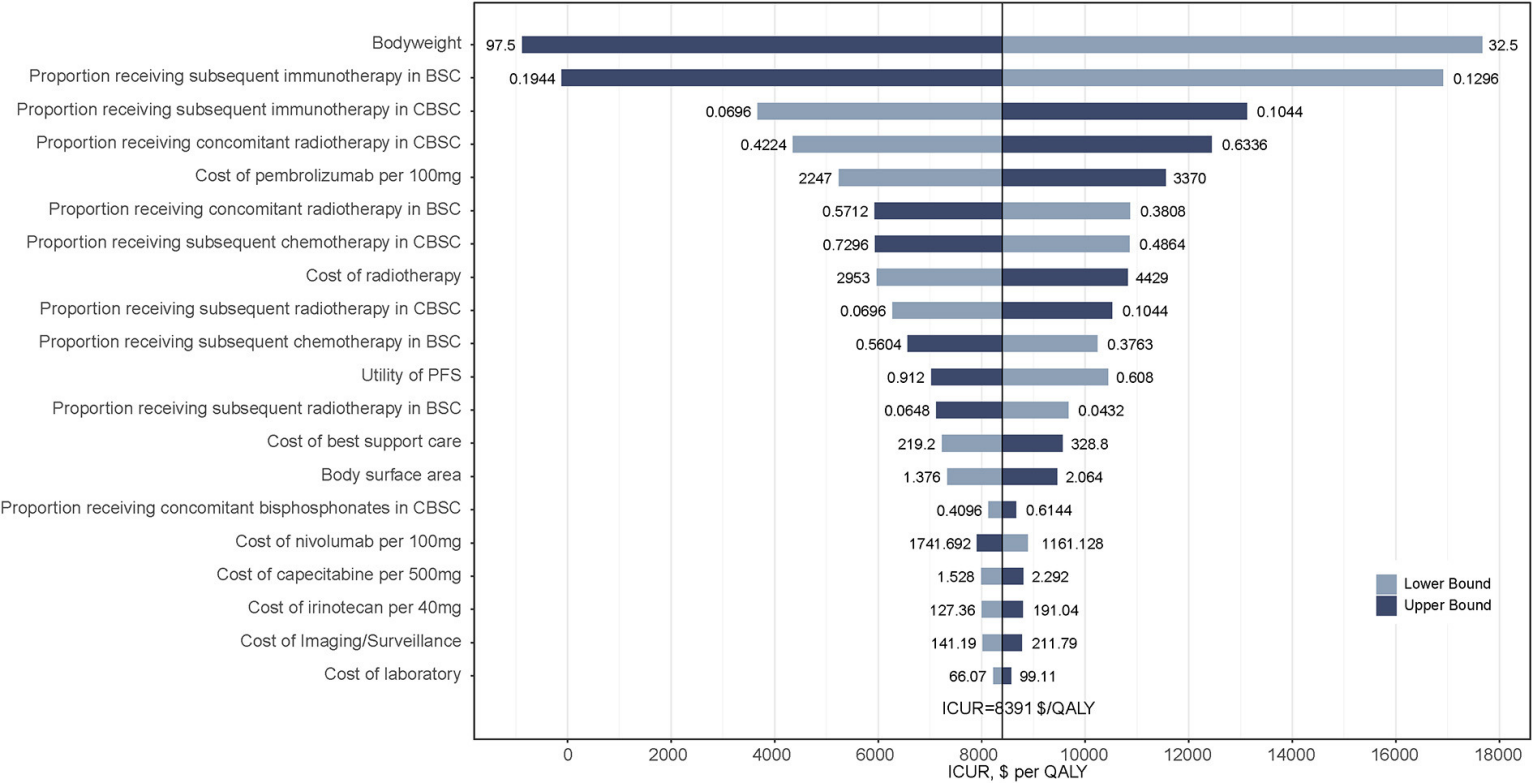
Tornado diagrams derived from the one-way sensitivity analysis of CBSC vs. BSC. Only the top 20 parameters that had the most influence on the results were shown. The black solid line indicates the ICURs. The dashed line indicates the WTP threshold in China ($36 007/QALY). CBSC, Capecitabine maintenance therapy plus best supported care group; BSC, best supported care; ICUR, incremental cost-utility ratios; QALY, quality-adjusted life-year.

Results from the one-way sensitivity analysis between two treatment strategies are presented in the tornado diagram (Figure 3). The results demonstrated that the body weight of patients, the proportion receiving subsequent immunotherapy in the BSC and CBSC groups, and the proportion receiving concomitant radiotherapy in the CBSC group played a vital role in the results of ICURs. Overall, varying the input parameters did not alter the conclusion that ICURs were lower than the WTP threshold.

**Figure 4:**
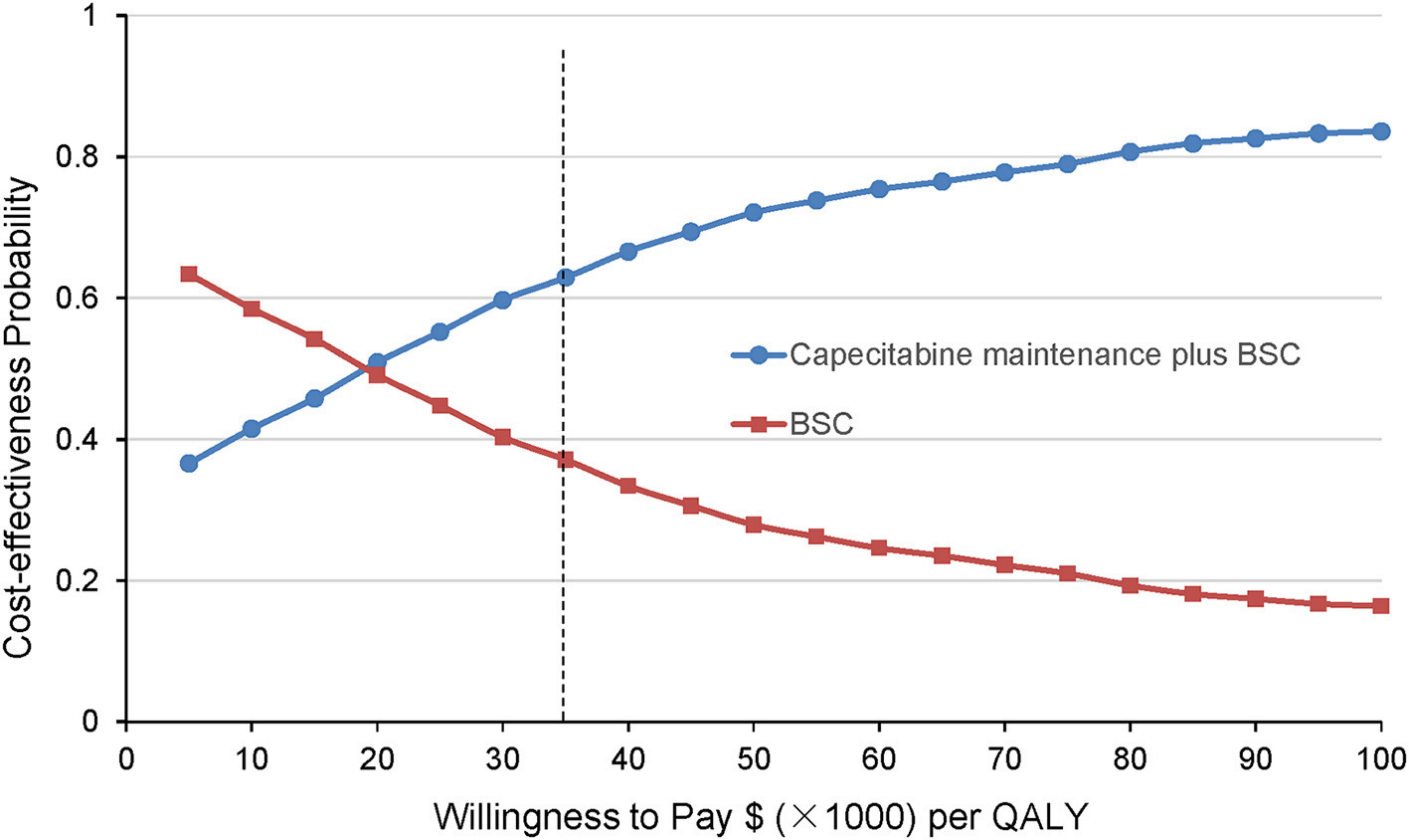
Cost-effectiveness acceptability curves derived from the probabilistic sensitivity analysis. The results are presented as probabilities that the treatment option is cost-effective at different willingness-to-pay thresholds. The black dashed line indicates the WTP threshold in China ($36 007/QALY). BSC, best-supported care; QALY, quality-adjusted life-year.

The cost-effectiveness acceptability curve by 10 000 Monte Carlo simulations revealed that compared with the BSC group, the probability of the CBSC group being cost-effective is 65.9% at the WTP threshold was $36 007/QALY (Figure 4).

## 4. Discussion

Most patients with early-stage NPC are responsive to the standard systemic radiotherapy and chemotherapy and are associated with a good prognosis, in addition to the mNPC (2). Furthermore, in the absence of further intensive therapy, most mNPC patients with disease recurrence will experience progression soon after first-line treatment. Although oncologists and patients are gradually interested in the promising immunotherapy maintenance option for recurrent or metastasis NPC based on the outcomes of the CAPTAIN-1st and JUPITER-02 clinical researches (12, 13), the high drug prices might be an important impediment to scale up. Low-dose maintenance chemotherapy was well tolerated with a low discontinuation rate and may be an attractive therapeutic intervention for the treatment of NPC. A recent clinical trial (NCT02460419) reported the efficacy and safety of capecitabine maintenance therapy in addition to systemic induction chemotherapy for patients with mNPC (26).

Based on the latest data released by the China's National Bureau of Statistic in 2021, the annual healthcare costs increased to ~7 trillion yuan ($1 trillion), carrying an enormous substantial economic burden on the health care system in China (36). Cost-effectiveness analysis based on randomized controlled trials (RCTs), that is, collection of patient-level cost data of treatments along with the measures of effectiveness is becoming increasingly common (26). This adds dimensions to interpret the results of RCTs and is designed to answer questions of health economic policy in addition to clinical benefits alone (15, 18). Therefore, we synthesized the latest evidence in the NCT02460419 trial and conducted the analysis to estimate the cost-effectiveness of the capecitabine maintenance therapy plus best supportive care, and best supportive care alone in the first-line treatment of newly diagnosed mNPC from the Chinese payers' perspective.

Overall, our analysis meets the CHEERS Checklist (Supplementary Table 5). According to the baseline analysis, after induction chemotherapy capecitabine maintenance treatment of mNPC was more cost-effective than induction chemotherapy alone at the WTP threshold of $36 007/QALY in China. As shown in Table 2, the ICUR was $8 391 per QALY in the baseline results, which was significantly lower than the WTP threshold. Meanwhile, the gained INHB in the CBSC group was positive at the threshold of $36 007 per QALY gained. The combined baseline results in the PFS state and across disease stages support the role of additional capecitabine in preventing disease progression was the primary driver of economic outcomes. All subgroups favored induction chemotherapy plus CBSC treatment due to the positive trend in INHB compared with the BSC treatment. After first-line induction chemotherapy, the stable disease response subgroup treated with CBSC had the highest probability to be cost-effective. According to the plasma EBV DNA status at baseline, patients with EBV DNA copy number > 4000 received CBSC treatment was more cost-effective than EBV DNA copy number ≤ 4000. Moreover, patients in the CBSC treatment group with lung or bone metastasis were more cost-effective than no present metastases, but those without liver metastasis were more cost-effective than present liver metastasis. It should be noted that the results of the subgroup analyses should be interpreted with caution owing to the lack of sufficient data and the heterogeneity of the population.

Finding on the one-way sensitivity analysis revealed that none of the key conclusions are changed by altering each parameter, which underlined the robustness of our Markov model. Further, the results of the probabilistic sensitivity analysis further demonstrate the stability of the model and the higher probability that the CBSC group was more cost-effective than the BSC group.

Most of the treatment-related AEs occurred in patients treated with capecitabine were manageable and no treatment-related deaths occurred. Therefore, capecitabine maintenance therapy does not introduce high additional possible costs for AEs treatment. In addition, a relatively appropriate price with a significant PFS benefit could be responsible for the capecitabine maintenance treatment after induction chemotherapy was more cost-effective compare to induction chemotherapy alone. Based on the results of the current study, capecitabine maintenance therapy could be an effective, safe, and cost-effective treatment, which appeared to be a promising new option for patients with mNPC.

To our knowledge, this is the first cost-effectiveness analysis to estimate the economic outcomes of the capecitabine maintenance therapy plus best supportive care in the first-line treatment of newly diagnosed mNPC from the Chinese payers' perspective. Though maintenance capecitabine has shown promising results for NPC in early trials (25, 26), further data for health and economic outcomes are needed before the drug can be accepted as a standard first-line treatment in the future. A previous cost-effectiveness analysis has shown that capecitabine and bevacizumab maintenance therapy for patients with metastatic colon cancer was not cost-effective at an ICER of $725 601/QALY from the US Medicare payer's perspective (41). Our study differs from this research that evaluated a combination regimen including capecitabine for metastatic colon cancer in the US. Another analysis evaluated the cost-effectiveness of metronomic capecitabine as adjuvant chemotherapy for locoregionally advanced NPC patients from the perspective of China (42). The results indicated that metronomic capecitabine as adjuvant chemotherapy is a cost-effective strategy, which obtained an ICER of $ 9669.99/QALY (42). Their results are consistent with ours, which revealed that capecitabine treatment was an effective and cost-effective choice for NPC in China.

Several strengths need to be emphasized. First, the NCT02460419 clinical trial was conducted in China and all enrolled patients were Chinese; thus, our study using price and clinical data in China provided an actionable and valuable evidence for policymakers, providers, and patients to make an optional decision. Second, in the present study, we used data from the life table to capture other causes of background mortality including cardiovascular diseases, allowing the model to better reflect reality. Third, multiple economic outcomes of prespecified subgroups in the NCT02460419 clinical trial were evaluated in our current analysis. Economic analysis of subgroups provides more precise information that may be helpful for clinicians and patients.

Some limitations of this study must be acknowledged. First, we did not include other standard immunotherapy-related first-line treatment options, e.g., camrelizumab or toripallimab plus chemotherapy due to the different inclusion criteria between the clinical trials. There is a lack of randomized controlled clinical trials testing maintenance immunotherapy vs. capecitabine maintenance therapy in patients with mNPC. Second, the OS data in the NCT02460419 clinical trial were not mature at the time of the analysis, which could have some impact on fitted survival data. Nevertheless, the long-term survival data of the two groups was extrapolated from the limited available survival curves from the NCT02460419 clinical trial using a specific mathematical model, thus, our results are unlikely to be strongly affected by immature OS data. Our analysis would be updated as new additional evidence becomes available. Third, because of the quality-of-life data have not been published in the NCT02460419 clinical trial, we used relevant data in the published literature. Subsequent one-way sensitivity analyses, therefore, indicate that changing the utility values would not alter our conclusions.

## 5. Conclusions

Based on the analysis, compared with BSC treatment, after induction chemotherapy, capecitabine maintenance treatment plus BSC as first-line treatment was a more cost-effective strategy for patients with newly diagnosed mNPC from the Chinese payers' perspective. Exploring treatment strategies tailored by the characteristics of the individual patient could be a way to improve the economic outcomes.

## Data availability statement

The original contributions presented in the study are included in the article/Supplementary material, further inquiries can be directed to the corresponding author.

## Author contributions

JiaH and KT: conceptualization. JiaH and XL: methodology. XS and XL: formal analysis and investigation. JiaH: writing—original draft preparation. JiaH and KT: writing—review and editing. JiaH and NC: funding acquisition. XS: resources. NC: supervision. All authors contributed to the article and approved the submitted version.
