# Supplementary material for: Cost-effectiveness analysis of capecitabine maintenance therapy plus best supportive care vs. best supportive care alone as first-line treatment of newly diagnosed metastatic nasopharyngeal carcinoma
Source: Front Public Health. 2023 Jan 26;10:1086393. doi: 10.3389/fpubh.2022.1086393 (PMC9911043; doi:10.3389/fpubh.2022.1086393)
Supplement: Supplementary file 1 [file Data_Sheet_1.PDF]

Supplement Tables and Figures

Supplementary Table 1: Patient Baseline Demographic and Clinical Characteristics

Supplementary Table 2: Drug Doses, Schedule, and Unit Price

Supplementary Table 3: Summary of Statistical Goodness-of-fit of Kaplan-Meier Survival Curves in the NCT02460419 trial

Supplementary Table 4: Background Mortality Rate

Supplementary Table 5: CHEERS Checklist

Supplementary Figure 1. Kaplan-Meier Curve Fitting and Extrapolation.

Supplementary Figure 2: Probabilities for Each State in Every Markov cycle

Supplementary Table 1: Patient Baseline Demographic and Clinical Characteristics

Characteristics	Capecitabine + BSC (N = 52) No. (%)	BSC alone (N = 52) No. (%)
**Age – years**		
Median age	45	49
≤45	29 (55.8)	24 (46.2)
＞45	23 (44.2)	28 (53.8)
**Sex**		
Male	43 (82.7)	41 (78.8)
Female	9 (17.3)	11 (21.2)
**ECOG performance score**		
0	13 (25.0)	11 (21.2)
1	39 (75.0)	41 (78.8)
**Histologic characteristics**		
Non-keratinizing		
Undifferentiated (type III)	51 (98.1)	49 (94.2)
Differentiated (type II)	1 (1.9)	0
Poorly differentiated	0	3 (5.8)
**Smoking status**		
Yes	10 (19.2)	15 (28.8)
No	42 (80.8)	37 (71.2)
**Stage**		
Primary metastases	33 (63.5)	28 (53.8)
Asynchronous metastases	19 (36.5)	24 (46.2)
**Metastasis type**		
Oligometastasis	16 (30.8)	15 (28.8)
Polymetastases	36 (69.2)	37 (71.2)
**Site of metastasis**		
Lung	17 (32.7)	20 (38.5)
Bone	33 (63.5)	30 (57.7)
Liver	20 (38.5)	11 (21.2)
**No. of first-line chemotherapy cycles**		
4-5	6 (11.5)	9 (17.3)
6	46 (88.5)	43 (82.7)
**Treatment response to first-line chemotherapy**		
Complete or partial response	43 (82.7)	42 (80.8)
Stable disease	9 (17.3)	10 (19.2)
**EBV DNA, copies/mL**		
≤4000	21 (40.4)	25 (48.1)
>4000	31 (59.6)	27 (51.9)

ECOG, Eastern Cooperative Oncology Group; EBV, Epstein-Barr virus.

The baseline demographic and clinical characteristics of the hypothetical patients in the model were derived from the relevant data of the NCT02460419 clinical trial.

Supplementary Table 2. Drug Doses, Schedule, and Unit Price

Drugs	Doses	Unit price ($)	Cost per cycle ($/3 wks.)	Reference
**Progression-free disease**				
Capecitabine	1000 mg/m² bid po on days 1-14	1.91/500mg	183.36	Local database
Paclitaxel	150 mg/m² IV on days 1	27.94/30mg	240.28	Local database
Cisplatin	60 mg/m² IV on day 1 every 3 weeks	3.00/30mg	10.32	Local database
Bisphosphonates	5mg IV on day 1 every 3 weeks	250.47/5mg	250.47	Local database
Radiotherapy-related cost		3690.64		Local database
**Progression disease**				
Capecitabine	1250 mg/m² bid po	1.91/500mg	266.83	Local database
Gemcitabine	1000 mg/m² IV on days 1 and 8 every 3 weeks	26.05/200mg	468.96	Local database
Docetaxel	30 mg/m² on days 1, 8, and 15 every 28 days	75.56/20mg	510.03	Local database
Irinotecan	100 mg/m² on days 1, 8, and 15 every 28 days	159.20/40mg	1790.95	Local database
Camrelizumab	200 mg IV every 2 weeks	458.93/200mg	688.40	Local database
Toripalimab	3 mg/kg every 2 weeks	142.01/80mg	426.02	Local database
Nivolumab	3 mg/kg every 2 weeks	1451.41/100mg	4354.23	Local database
Pembrolizumab	10 mg/kg every 2 weeks	2808.46/100mg	25276.18	Local database
Radiotherapy-related cost		3690.64		Local database

Supplementary Table 3: Summary of Statistical Goodness-of-fit of Kaplan-Meier Survival Curves in the NCT02460419 trial

	Exponential	Weibull	Log-logistic	Lognormal	Gompertz
**CBSC PFS curve**					
AIC	-229.0381	-336.6797	-374.8541	-363.6529	56.5487
BIC	-224.1063	-329.282	-367.4564	-356.2552	63.94643
**BSC PFS curve**					
AIC	-109.97807	-218.32037	-252.52295	-250.39301	-87.59533
BIC	-105.0463	-210.9226	-245.1252	-242.9953	-80.1976
**CBSC OS curve**					
AIC	-349.0086	-352.4075	-378.8661	-365.3678	-73.41731
BIC	-343.9869	-344.8749	-371.3335	-357.8352	-65.88474
**BSC OS curve**					
AIC	-335.5018	-340.0031	-376.2531	-367.6451	-354.1761
BIC	-330.7884	-332.9330	-369.1830	-360.5750	-347.1060

CBSC, capecitabine maintenance therapy plus best supported care; BSC, best supportive care; OS, overall survival; PFS, progression-free survival; AIC, Akaike's information criterion; BIC, Bayesian information criterion

Supplementary Table 4: Background Mortality Rate

Age	Background mortality rate	Age	Background mortality rate	Age	Background mortality rate
23	0.00054	50	0.00363	77	0.04970
24	0.00056	51	0.00374	78	0.05466
25	0.00058	52	0.00397	79	0.06025
26	0.00057	53	0.00440	80	0.07162
27	0.00059	54	0.00497	81	0.07499
28	0.00061	55	0.00517	82	0.08228
29	0.00068	56	0.00562	83	0.08934
30	0.00070	57	0.00607	84	0.09852
31	0.00077	58	0.00679	85	0.10516
32	0.00081	59	0.00764	86	0.11221
33	0.00083	60	0.00850	87	0.12213
34	0.00094	61	0.00934	88	0.13448
35	0.00103	62	0.01033	89	0.14546
36	0.00106	63	0.01106	90	0.16220
37	0.00114	64	0.01293	91	0.16954
38	0.00121	65	0.01411	92	0.18356
39	0.00134	66	0.01463	93	0.18787
40	0.00151	67	0.01708	94	0.18915
41	0.00155	68	0.01847	95	0.19821
42	0.00182	69	0.02167	96	0.19900
43	0.00189	70	0.02525	97	0.18581
44	0.00207	71	0.02638	98	0.18027
45	0.00231	72	0.03047	99	0.22825
46	0.00236	73	0.03304	100	0.66667
47	0.00254	74	0.03675		
48	0.00311	75	0.04067		
49	0.00327	76	0.04132		

The background mortality rate in the model was estimated based on the China life table. The start age was assumed as 45 years old in the CBSC group and 49 years old in the BSC group, respectively, which was based on the median age in the baseline characteristic.

Supplementary Table 5: CHEERS Checklist

Section/item	Item No	Recommendation	Reported?
**Title and abstract**			
Title	1	Identify the study as an economic evaluation or use more specific terms such as "cost-effectiveness analysis", and describe the interventions compared.	**Yes**
Abstract	2	Provide a structured summary of objectives, perspective, setting, methods (including study design and inputs), results (including base case and uncertainty analyses), and conclusions.	**Yes**
**Introduction**			
Background and objectives	3	Provide an explicit statement of the broader context for the study. Present the study question and its relevance for health policy or practice decisions.	**Yes**
**Methods**			
Target population and subgroups	4	Describe characteristics of the base case population and subgroups analyzed, including why they were chosen.	**Yes**
Setting and location	5	State relevant aspects of the system(s) in which the decision(s) need(s) to be made	**Yes**
Study perspective	6	Describe the perspective of the study and relate this to the costs being evaluated.	**Yes**
Comparators	7	Describe the interventions or strategies being compared and state why they were chosen.	**Yes**
Time horizon	8	State the time horizon(s) over which costs and consequences are being evaluated and say why appropriate.	**Yes**
Discount rate	9	Report the choice of discount rate(s) used for costs and outcomes and say why appropriate.	**Yes**
Choice of health outcomes	10	Describe what outcomes were used as the measure(s) of benefit in the evaluation and their relevance for the type of analysis performed.	**Yes**
Measurement of effectiveness	11a	*Single study-based estimates*: Describe fully the design features of the single effectiveness study and why the single study was a sufficient source of clinical effectiveness data.	**Yes**

	11b	*Synthesis-based estimates*: Describe fully the methods used for identification of included studies and synthesis of clinical effectiveness data.	**N/A**
Measurement and valuation of preference based outcomes	12	If applicable, describe the population and methods used to elicit preferences for outcomes.	**N/A**
Estimating resources and costs	13a	*Single study-based economic evaluation*: Describe approaches used to estimate resource use associated with the alternative interventions. Describe primary or secondary research methods for valuing each resource item in terms of its unit cost. Describe any adjustments made to approximate to opportunity costs.	**N/A**
	13b	*Model-based economic evaluation*: Describe approaches and data sources used to estimate resource use associated with model health states. Describe primary or secondary research methods for valuing each resource item in terms of its unit cost. Describe any adjustments made to approximate to opportunity costs.	**Yes**
Currency, price date, and conversion	14	Report the dates of the estimated resource quantities and unit costs. Describe methods for adjusting estimated unit costs to the year of reported costs if necessary. Describe methods for converting costs into a common currency base and the exchange rate.	**Yes**
Choice of model	15	Describe and give reasons for the specific type of decision-analytical model used. Providing a figure to show the model structure is strongly recommended.	**Yes**
Assumptions	16	Describe all structural or other assumptions underpinning the decision-analytical model.	**Yes**
Analytical methods	17	Describe all analytical methods supporting the evaluation. This could include methods for dealing with skewed, missing, or censored data; extrapolation methods;	**Yes**

		methods for pooling data; approaches to validate or make adjustments (such as half cycle corrections) to a model; and methods for handling population heterogeneity and uncertainty.	
**Results**			
Study parameters	18	Report the values, ranges, references, and, if used, probability distributions for all parameters. Report reasons or sources for distributions used to represent uncertainty where appropriate. Providing a table to show the input values is strongly recommended.	**Yes**
Incremental costs and outcomes	19	For each intervention, report mean values for the main categories of estimated costs and outcomes of interest, as well as mean differences between the comparator groups. If applicable, report incremental cost-effectiveness ratios.	**Yes**
Characterizing uncertainty	20a	*Single study-based economic evaluation*: Describe the effects of sampling uncertainty for the estimated incremental cost and incremental effectiveness parameters, together with the impact of methodological assumptions (such as discount rate, study perspective).	**N/A**
	20b	*Model-based economic evaluation*: Describe the effects on the results of uncertainty for all input parameters, and uncertainty related to the structure of the model and assumptions.	**Yes**
Characterizing heterogeneity	21	If applicable, report differences in costs, outcomes, or cost-effectiveness that can be explained by variations between subgroups of patients with different baseline characteristics or other observed variability in effects that are not reducible by more information.	**N/A**
**Discussion**			
Study findings, limitations, generalizability, and current	22	Summarize key study findings and describe how they support the conclusions reached. Discuss limitations and the generalizability	**Yes**

knowledge		of the findings and how the findings fit with current knowledge.	
**Other**			
Source of funding	23	Describe how the study was funded and the role of the funder in the identification, design, conduct, and reporting of the analysis. Describe other non-monetary sources of support.	**Yes**
Conflicts of interest	24	Describe any potential for conflict of interest of study contributors in accordance with journal policy. In the absence of a journal policy, we recommend authors comply with International Committee of Medical Journal Editors recommendations.	**Yes**

Supplementary Figure 1. Kaplan-Meier Curve Fitting and Extrapolation.

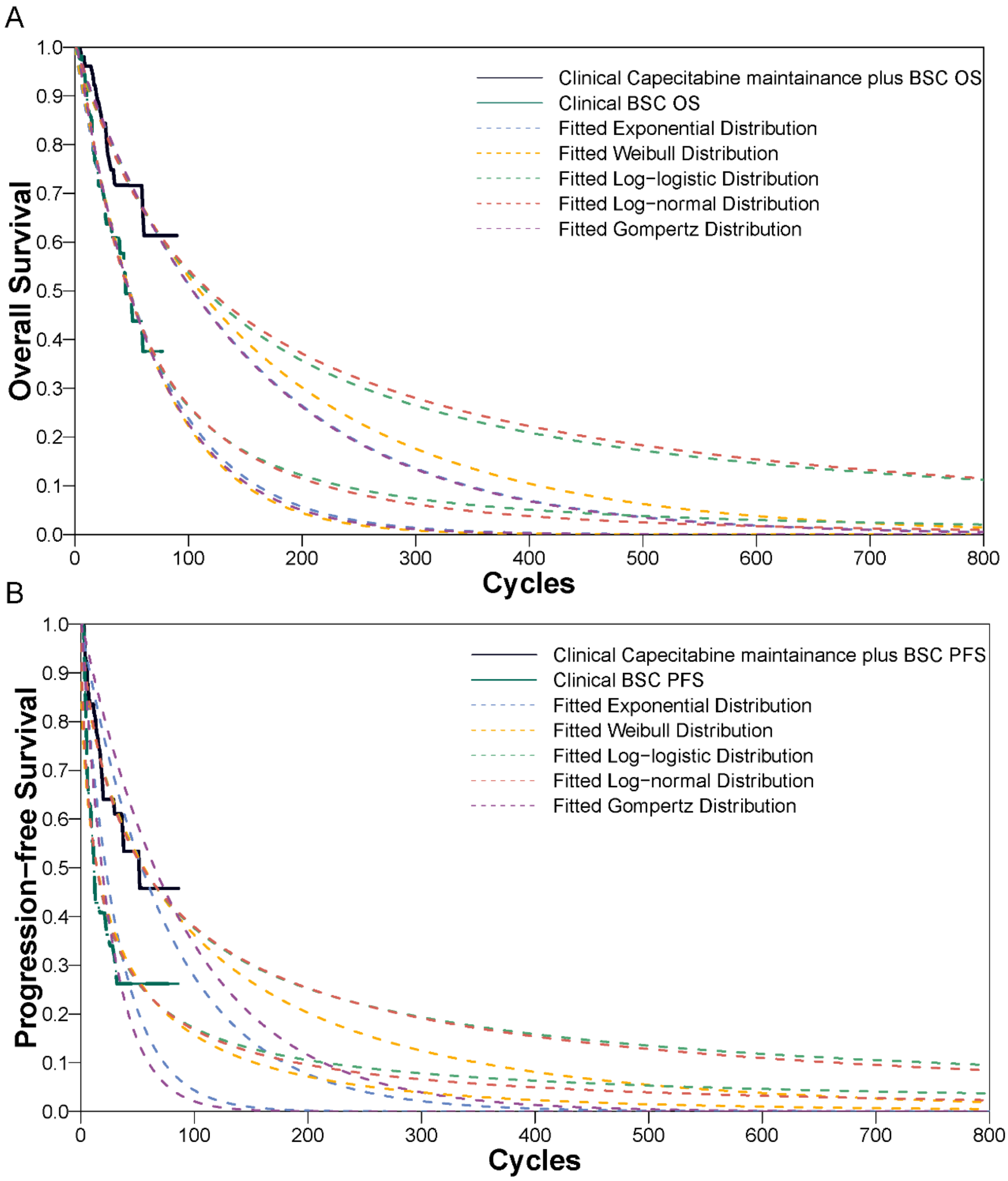

(A) Fitting and extrapolation of the overall survival curves; (B) Fitting and extrapolation of the progression-free survival curves

BSC, best supportive care; OS, overall survival; PFS, progression-free survival

Supplementary Figure 2: Probabilities for Each States in Every Markov cycle

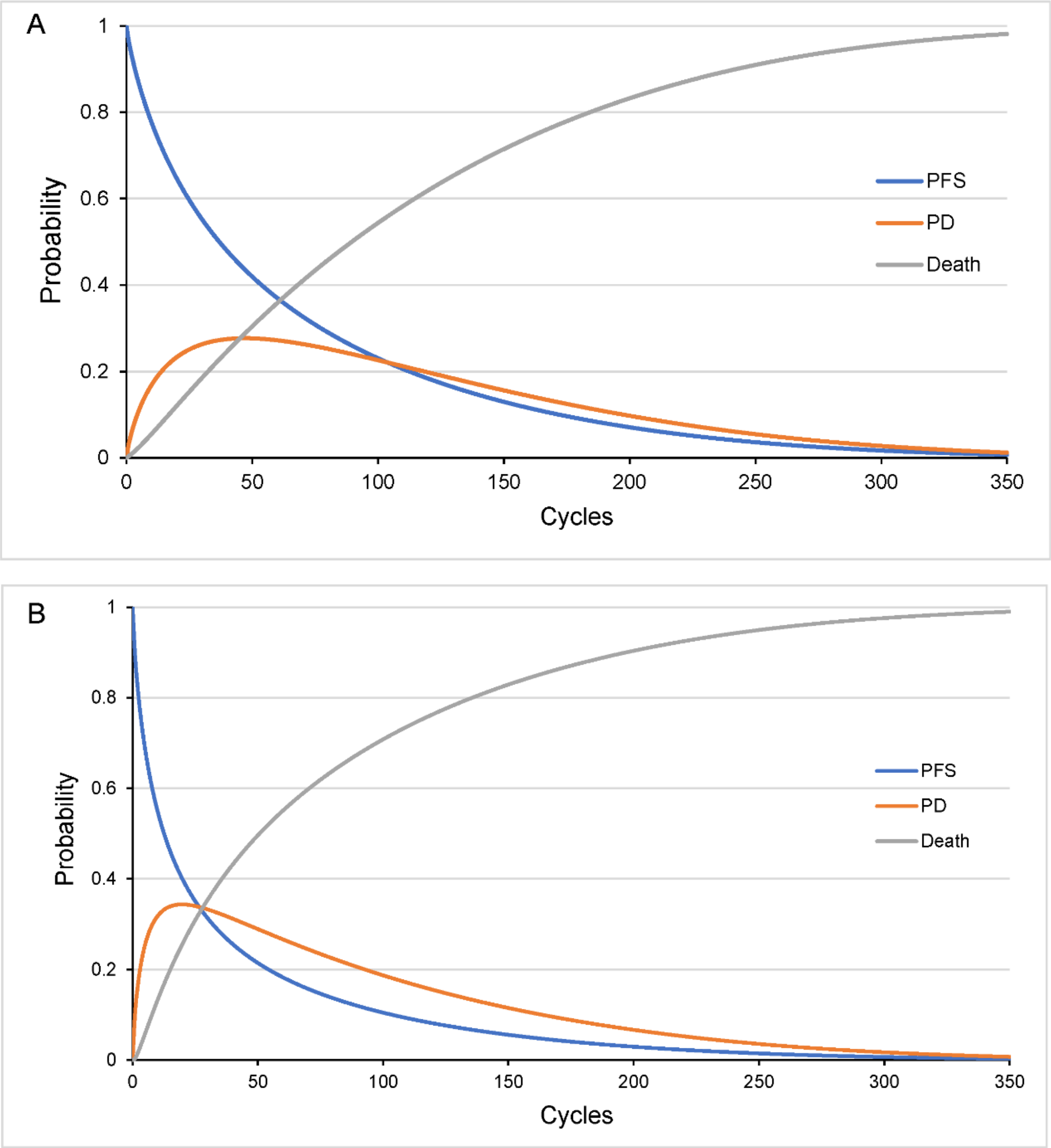

(A) Capecitabine maintenance therapy plus best supported care group; (B) Best supported care group.

OS, overall survival; PFS, progression-free survival state; PD, Progression disease state
